# Nonuniform structural properties of wings confer sensing advantages

**DOI:** 10.1098/rsif.2022.0765

**Published:** 2023-03-22

**Authors:** Alison I. Weber, Mahnoush Babaei, Amanuel Mamo, Bingni W. Brunton, Thomas L. Daniel, Sarah Bergbreiter

**Affiliations:** ^1^ Department of Biology, University of Washington, Seattle, WA, USA; ^2^ Department of Aerospace Engineering and Engineering Mechanics, University of Texas at Austin, Austin, TX, USA; ^3^ Department of Materials Science and Engineering, University of Washington, Seattle, WA, USA; ^4^ Department of Mechanical Engineering, Carnegie Mellon University, Pittsburgh, PA, USA

**Keywords:** flexible wings, sensing, flapping flight, finite element analysis

## Abstract

Sensory feedback is essential to both animals and robotic systems for achieving coordinated, precise movements. Mechanosensory feedback, which provides information about body deformation, depends not only on the properties of sensors but also on the structure in which they are embedded. In insects, wing structure plays a particularly important role in flapping flight: in addition to generating aerodynamic forces, wings provide mechanosensory feedback necessary for guiding flight while undergoing dramatic deformations during each wingbeat. However, the role that wing structure plays in determining mechanosensory information is relatively unexplored. Insect wings exhibit characteristic stiffness gradients and are subject to both aerodynamic and structural damping. Here we examine how both of these properties impact sensory performance, using finite element analysis combined with sensor placement optimization approaches. We show that wings with nonuniform stiffness exhibit several advantages over uniform stiffness wings, resulting in higher accuracy of rotation detection and lower sensitivity to the placement of sensors on the wing. Moreover, we show that higher damping generally improves the accuracy with which body rotations can be detected. These results contribute to our understanding of the evolution of the nonuniform stiffness patterns in insect wings, as well as suggest design principles for robotic systems.

## 1. Introduction

To execute precise movements, animals and robotic systems alike rely on sensory feedback conveying information about their environment and their own body conformation. Mechanosensory information processing is especially important in movement control. From the capacity to detect pressure and vibration for manipulation tasks to the crucial role of proprioception in locomotion, living systems deploy diverse sensory mechanisms for encoding mechanical information. In all of these systems, sensors are embedded in complex, three-dimensional structures whose geometry (size and shape) and material properties (stiffness, damping, density) transform forces into deformations. Therefore, the information encoded by mechanosensors is not only determined by properties of the sensors themselves but is fundamentally shaped by the properties of the structure in which those sensors are embedded.

The interplay between structure and sensing is particularly important in the case of flight. As insects beat their wings, the wings undergo dramatic deformations due to a combination of inertial and aerodynamic forces. Information about these deformations is encoded by a population of strain-sensitive structures, called campaniform sensilla, sparsely arrayed over the surface of the wing [1]. Information about wing deformation is used to guide behaviour during flight [2]. The characteristics of wing deformations are determined both by how the animal moves its wings as well as by the structural properties of the wings. Whereas the impacts of wing structure on aerodynamic performance are well studied in both biological and engineered systems [3–14], the impacts on sensory performance are relatively unknown.

Previous work on the sensory functions of wings showed that information about body rotation can be accurately detected in a very small (fewer than 10) population of optimally placed sensors [15]. Additional work showed that wing stiffness significantly impacts sensory performance, with small changes in wing stiffness sometimes resulting in abrupt transitions in sensory performance and optimal sensor placement [16]. However, previous work has been limited by relying on highly idealized analytical wing models (Euler–Lagrange models). These models are unable to capture the rich morphological characteristics of real insect wings or the diverse array of structural properties one might consider in designing an engineered system, such as complex shape and nonuniform material properties. These properties will necessarily impact wing bending and therefore will impact the sensory signals encoded by mechanosensors in the wing. Properties such as shape and stiffness significantly impact wing deformations, the resulting aerodynamic forces and an animal’s behavioural performance [4,17–19]. Models based on the finite-element method (FEM) support examination of more realistic wing features, such as wing geometry, venation patterns, damping and nonuniform stiffness [5,8,17,20–22]. However, these models have not yet been used to examine sensing. Nonuniform structural properties will result in more complex spatio-temporal patterns of wing strain and could significantly impact sensing performance and optimal sensing strategies.

In this work, we examine the sensory consequences of wing properties, namely wing stiffness gradients and damping properties, that could not be examined with more simplified models. We employ computational approaches to examine how sensing performance depends on these properties. To do so, we first develop a series of flapping wing models using the finite element method, simulating wings with a range of stiffness and damping properties. For each of these wings, we encode information about strain in a population of optimized neural-inspired sensors. We then evaluate whether inertial perturbations such as body rotations, which induce subtle changes in wing bending, can be detected via simple decoding mechanisms. We show that nonuniform stiffness improves sensing performance across a range of other model properties. Further, increased damping generally increases sensing performance and reduces the need to fine-tune other properties of the wing to maximize performance. Thus, in addition to the advantages of nonuniform stiffness in the production of flight forces, we demonstrate here additional advantages to sensing. These results provide an inroad to our understanding of the joint evolution of sensing and actuation mediated by the structural dynamics of wings and additionally suggest possible strategies for improved design of engineered flight systems.

## 2. Methods

### 2.1. Overview

Our goal is to assess how sensing performance depends on the structural properties of flapping wings. To examine this, we first simulate the complex spatio-temporal patterns of strain that arise in flexible, flapping wings using a finite element analysis (FEA) model. We then encode this strain by simulating neural-inspired spiking sensors in a dense grid over the surface of the wing. These simulations are modelled after strain sensors (campaniform sensilla) found on the wings of flying insects. Finally, we use a previously established optimization method [23] to identify the locations of a small population of sensors from which information about wing rotation can best be read out. We perform this series of steps for multiple wings with different structural properties—namely, different average stiffness, stiffness gradient and damping ratio—to determine how sensing accuracy and optimal sensor locations depend on these properties.

A COMSOL file that implements the FEA model as well as multiple example simulation results are available at https://doi.org/10.5061/dryad.fxpnvx0wq [24]. MATLAB code that implements strain encoding in neural-inspired sensors and sensor placement optimization can be found at https://github.com/aiweber/optimal_sensing_FEAwing.

### 2.2. Finite element model

An FEA model is created in COMSOL Multiphysics 5.6 software to study the dynamics of a flapping wing and the effects of various structural properties on the complex spatio-temporal strain distribution on the wing. The finite element model is developed for a geometrically simplified wing that shares several characteristics with the wings of the hawkmoth *Manduca sexta* (e.g. size and flapping frequency). In contrast to earlier work that relied on analytical models based on the Euler–Lagrange equations, finite element models provide the versatility to study a variety of complex properties of real insect wings, such as irregular shapes, inhomogeneous material properties and anisotropies [17,22,25–27].

**Figure 1.**
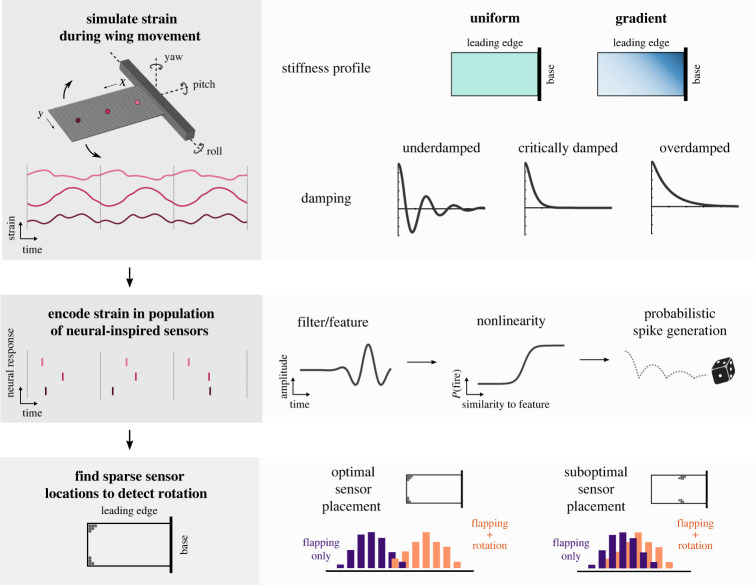
Assessing sensing performance on wings with different structural properties. Top: we use a finite element model to simulate strain in flapping wings with different structural properties. Wings may be uniformly stiff (green) or follow a stiffness gradient (shaded blue), from stiffest at a corner of the wing base to most compliant at the opposite corner on the wing tip. Wings may be either overdamped, critically damped or underdamped. The output of a simulation for a given wing is strain over time at each location on the wing (e.g. light, medium and dark pink lines show example strain for a uniform stiffness wing of 0.3 GPa). Middle: strain at each location is encoded in a neural-inspired spiking sensor. The strain signal at each location is convolved with a filter, representing a temporal feature of strain the sensor is most sensitive to. Filtered strain is then passed through a sigmoidal function to determine the probability of firing a spike, from which spikes are probabilistically generated. Coloured vertical lines indicate spike times at locations with corresponding colours above. Bottom: optimization methods are used to identify a sparse set of sensor locations (grey dots) from which information about rotation can best be decoded. Optimal sensor placement will result in greater separation between responses in flapping wings (purple) and responses in wings flapping while undergoing rotation (orange).

Here we focus on a few specific aspects of wing structure and model the wing as a single 25 mm × 50 mm rectangular plate with a thickness of 127 μm (figure 1). The thickness is considered to be uniform throughout the plate and the effect of venation is neglected in the simulations. The material properties used in the FEA model are a density of 1180 kg m^−3^ and Poisson’s ratio of 0.35. Based on prior experimental studies [17,28], we considered the wing stiffness to be linearly elastic. It is possible that, under very large deformations, nonlinear behaviours may arise. We currently do not have experimental information regarding possible nonlinearities. The impact of flexural stiffness on the strain patterns is studied by varying Young’s modulus (*E*) of the plate. The stiffness could also be changed by varying geometrical features of the wing, e.g. introducing nonuniform thickness throughout the wing. However, in this work we examine changes in stiffness only using the Young’s modulus. The range of explored values for the modulus spans two orders of magnitudes around 3 GPa, corresponding to the average of experimentally measured Young’s modulus for *Manduca sexta* [28].

Experimental evidence from prior studies shows that the flexural stiffness of insect wings is not uniform. Specifically, flexural stiffness decays logarithmically, diagonally across the wing from the leading edge base to the trailing edge tip [17]. To understand the effect of this nonuniformity on the capability of the wing to detect rotations, two groups of wings with a (i) uniform and (ii) nonuniform distribution of Young’s modulus are studied.

In the first group of wings, a constant Young’s modulus (*E*_*c*_) is assigned throughout the plate. In the second group of wings, a logarithmic decay in flexural stiffness of the flapping wing is prescribed through a spatially variable Young’s modulus *E*_(*X*,*Y*)_:

2.1 $$E_{\left(X,Y\right)}=E_{c}\frac{10^{-m_{x}X}\times 10^{-m_{y}Y}}{\bar{10^{-m_{x}x}\times 10^{-m_{y}y}}},$$

where *X* and *Y* are the positions on the wing surface, *x* and *y* are the entire set of coordinates on the wing and *m*_*x*_ and *m*_*y*_ are the logarithmic decline rates along *x* and *y*, respectively. The overbar in the denominator indicates the mean value of function $10^{-m_{x}x}\times 10^{-m_{y}y}$ within the wing. Equation (2.1) ensures a spatial distribution of stiffness around a mean value of *E*_*c*_. To achieve two orders of magnitude difference between the minimum and maximum *E* in the plate (approximately what is observed in hawkmoth wings [17]), a value of 26.67 is applied to the decline rates in both directions, *m*_*x*_ and *m*_*y*_.

Damping can have significant effects on the dynamic response and the strain patterns generated due to flapping and rotation. Here, we use viscous damping applied isotropically in the form of volumetric forces to the wing. The damping force is defined as *F* = −*c***V**, where **V** is the velocity vector at each time instant in all three directions and *c* is the damping coefficient of the structure defined as

2.2 $$c=2\zeta m\omega_{n},$$

where *ζ* is the damping ratio, *m* is the mass of the wing and *ω*_*n*_ is the natural frequency of the structure. Values of *ζ* = 0.2, 1.0 and 2.0 are chosen to represent a broad range of damping cases, i.e. underdamped, critically damped and overdamped, respectively. The resonance frequency for the first vibrational mode of the system is calculated using the eigenfrequency study in COMSOL. The eigenfrequency analysis is conducted for each stiffness set-up, and the results are used to determine the damping coefficient of the wing. (See electronic supplementary material, table S2, for selected results from this analysis.)

Second-order serendipity elements are used to (i) achieve more accurate results compared with first-order elements while (ii) lowering the computational cost compared with the second-order Lagrange elements. To achieve high-quality meshing, a structured quadrilateral mesh is generated on the largest surface of the wing and is swept through the thickness of the wing to create hexahedral meshing in the domain. To determine the size of the mesh, we conducted two sets of convergence analyses: (i) eigenfrequency analysis and (ii) tip displacement during flapping of the wing (electronic supplementary material, figure S4). Using 25 × 50 structured hexahedral elements balances the accuracy and the computational cost of simulations by keeping the error lower than 1% for both cases.

Flapping and rotation about different axes are applied through a rigid frame similar to a gyroscopic structure in shape (electronic supplementary material, figure S2). A constant rotation is applied about a single axis (either roll, pitch, or yaw) at a velocity of 1 rad s^−1^. Using the results from prior studies [29], the flapping pattern of the wing is defined in terms of two harmonic parts: (i) amplitude *A*_1_ = *π*/12 and frequency *f*_1_ = 25 Hz and (ii) amplitude *A*_2_ = *π*/60 and frequency *f*_2_ = 50 Hz:

2.3 $$\phi (t)=A_{1}\sin(2\pi f_{1}t)+A_{2}\sin(2\pi f_{2}t).$$

To numerically solve for the wing deformation, we use the implicit method of backward differentiation formula (BDF) with a time step of 2 × 10^−4^ s. To ensure stability of the solver, the maximum order of the BDF is set to 1. In addition, the rotation rate (when applied) is increased from 0 rad s^−1^ to its maximum value (1 rad s^−1^) using a ramp function over the course of one flapping cycle to ensure convergence and avoid dynamics resulting from an abrupt onset of motion (electronic supplementary material, figure S3).

### 2.3. Strain encoding in neural-inspired spiking sensors

The local normal strain in the direction of the wing span (*x*) is then encoded by a dense grid of neural-inspired sensors, from which an optimal subset will be chosen to assess sensing performance, as in previous work [16]. These model neurons reflect observed properties of sensory neurons innervating campaniform sensilla, strain-sensitive structures arrayed over wing veins [30]. At each node across the 25 × 50 grid of elements (corresponding to every 1 mm), strain is converted to a series of temporally sparse all-or-none sensing events using a linear–nonlinear model, a common model of neural responses [31]. In this model, strain is first convolved with a feature that represents the temporal pattern of strain to which the sensor is most sensitive. The filtered strain is then converted to probability of firing (i.e. triggering a response), *P*(fire), via a nonlinear function, such that greater similarity to the feature results in a higher probability of firing. This function reflects the sensor’s sensitivity to the feature of interest, with high threshold and steep slopes conferring greater selectivity than low thresholds and shallow slopes.

The shapes of the linear filter and nonlinearity are computed from previous electrophysiological recordings of responses in mechanosensors of the wing nerve [30]. The filter *f* is defined as a decaying sinusoidal function:

2.4 $$f\;(t,\omega ,\tau ,\delta )=\cos(2\pi \omega (t+\tau ))\cdot \exp\left(\frac{-{\left(t+\tau \right)}^{2}}{\delta^{2}}\right),$$

where *ω* is the frequency of the filter, *τ* is the time offset to the peak and *δ* is the decay time. In this work, *ω* = 1/(2*π*) ms^−1^, *τ* = 5 ms and *δ* = 4 ms. The sigmoidal nonlinearity is given by

2.5 $$N(g_{t},\alpha ,\beta )=\frac{1}{1+\exp(-\alpha (g_{t}-\beta ))},$$

where *g*_*t*_ is the filtered strain at time *t*, *α* is the slope parameter and *β* is the threshold parameter where the function reaches half-maximum. We hold *α* constant at 5 × 10^5^. For results in the main text, the threshold *β* is held constant at 1 × 10^−4^. We also tested the effects of varying the threshold from 1 × 10^−10^ to 1 because the neural threshold substantially impacted results in previous work [16]. In the present study, altering the neural threshold does not alter conclusions. (See the electronic supplementary material for full results of varying the neural threshold.)

We then generate spikes probabilistically from the output of the linear–nonlinear encoding. The sensor spikes if the probability of firing exceeds a random draw from a standard uniform distribution. We manually impose an absolute refractory period of 15 ms between spikes. This is not intended to represent the actual absolute refractory period of mechanosensors, but rather to empirically match observations from previous experimental work that each sensor fires only one to two spikes per wingbeat [32,33]. For each unique wing and body rotation condition, we generate 100 spiking responses for each sensor. Although the simulated strain is identical at a given location across these repetitions, stochasticity in generating the spiking response results in varied response times at a given location across multiple wingbeats.

### 2.4. Sensor placement optimization

Our objective in sensor placement optimization is to determine the placement of a small number (10) of neural-inspired sensors which can be used to determine whether or not the insect is rotating. We simulate spiking data as described above for two cases: one where a wing is flapping and one where a wing is flapping and rotating (in the roll, pitch or yaw axis). For both of these cases, we determine the time to first spike within each wingbeat with 0.1 ms precision and use only this spike timing information to classify the data. For wingbeats where no spike is elicited, we designate the spike time as zero, as in previous work [16]. Data are standardized in this optimization step, but the original (non-standardized) data are used to evaluate accuracy.

To determine optimal sensor locations, we use a previously developed method called *sparse sensor placement optimization for classification* (SSPOC) [34]. This method first uses dimensionality reduction (principal component analysis, in this case) to find a lower-dimensional subspace that captures important features of the data. We then use a linear discriminant analysis (LDA) to find the projection vector *w* that maximally separates the classes of our data in this subspace. Finally, we use elastic net regularization (which linearly combines lasso, or *L*_1_, regularization and ridge, or *L*_2_, regularization) to solve for a sparse set of sensors *s* that can reconstruct the projection vector *w*. We solve:

2.6 $$s=\underset{s^{\prime }}{\arg\;\min}[\lambda \|s^{\prime }{\|}_{1}+(1-\lambda )\|s^{\prime }{\|}_{2}]\;\text{subject to }\Psi^{T}s^{\prime }=w,$$

where *s*′ is a vector of sensor weights (*n* × 1, with many near-zero entries), *s* is the vector of optimized sensor weights, Ψ is the low-dimensional basis (*n* × *m*, *m* < *n*), *w* is the projection vector in the low-dimensional subspace (*m* × 1) and *λ* determines the balance between lasso and ridge regularization. We set *m* = 3 and *λ* = 0.9. We use the cvx package to solve this optimization problem (http://cvxr.com/cvx/) [35,36].

### 2.5. Performance evaluation

Ninety per cent of the data is randomly selected to be used as a training set for the optimization, and 10% is held out as test data to evaluate accuracy. For straightforward comparison across conditions, we consistently use the top 10 sensors (i.e. sensors with the largest weights in *s*) to assess classification accuracy. In previous work, we find that 10 sensors are typically enough to achieve near-peak accuracy without including a large number of extraneous sensors [16]. This is also a reasonable number of sensors for engineered systems to ensure reduced noise and latency along with higher classification accuracies [37]. LDA is again used to find the best projection vector *w*_*c*_ for the non-standardized test data for only the top 10 sensors. A decision boundary is drawn at the mean of the two condition centroids.

## 3. Results

Our goal is to assess how the structural properties of wings, namely the stiffness gradient and damping, affect an insect’s ability to detect behaviourally relevant perturbations to the wing. For simplicity, we focus on the task of identifying an insect’s body rotation from the strains in its flapping wing. Rotations at realistic speeds produce perturbations in wing strain that are several orders of magnitude smaller than strain produced by wing flapping [15], making this a challenging detection task.

Examples of the results obtained at various points throughout our process for sensor placement optimization are shown in figure 1. To evaluate detection accuracy for a wing, we first simulate wing bending as the wing undergoes a periodic flapping motion using the finite element model (figure 1, top). We calculate the time-varying strain at each location on a dense grid along the wing (1 mm spacing; example locations shown in figure 1, top: dark, medium and light pink lines). Next, we encode this strain in a population of neural-inspired sensors (figure 1, middle), where sensor properties are chosen to reflect the characteristics of mechanosensitive neurons in insect wings (see Methods for details). Finally, we identify an optimal subset of 10 sensor locations to detect wing perturbations using the SSPOC approach (figure 1, bottom) [23].

We repeat this approach for different model wings with varying structural properties to assess the impact of these properties on sensing accuracy. We simulate wings across a range of stiffness values, of both uniform stiffness and logarithmically decaying stiffness, the latter reflecting stiffness gradients observed in insect wings [28]. For each average stiffness and stiffness gradient, we simulate wings that are underdamped, critically damped and overdamped. We separately assess the ability to detect rotation about the roll, pitch and yaw axes in each of these wings.

**Figure 2.**
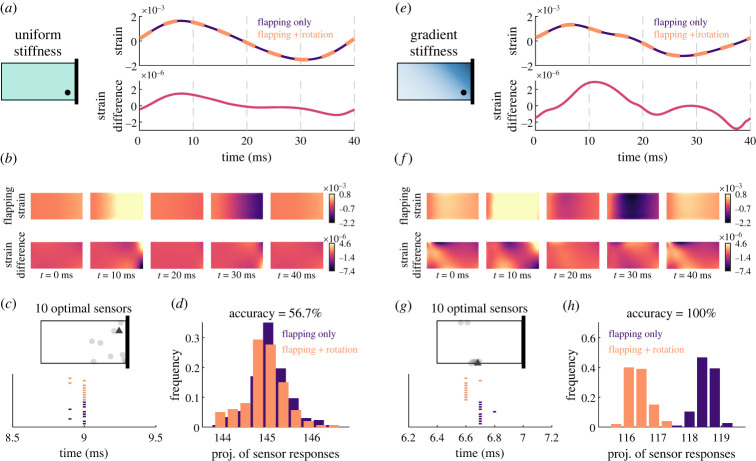
Simulated strain and optimal sensor locations for two example wings, one with uniform stiffness and one with a stiffness gradient. (*a*) Top: strain over the course of a single wingbeat for a uniform stiffness wing that is undergoing flapping only (purple) or flapping along with rotation about the yaw axis (orange). Bottom: strain difference between flapping only and flapping with rotation. (*b*) Top: spatial patterns of strain at select time points for a flapping wing (without rotation). Bottom: spatial pattern of the strain difference between the flapping only and flapping with rotation cases at select time points. (*c*) Top: optimal locations for 10 sensors to detect rotation about the yaw axis. Bottom: sensor response times for the single best sensor, indicated by a dark grey triangle in the wing schematic above, on trials when either the wing was only flapping (purple) or flapping while also rotating (orange). (*d*) Projection of the optimal sensor responses onto the vector that maximally separates the two groups. Greater separation between the two distributions leads to greater accuracy in determining whether the wing was rotating. (*e*–*h*) Same as (*a*–*d*) for a wing with a stiffness gradient, with average stiffness (3.0 GPa) matched to the wing in (*a*).

To start, we focus on the impacts of nonuniform stiffness, a common property of insect wings that has been shown to be favourable for aerodynamic performance [5]. Regardless of a wing’s stiffness gradient, strain produced by flapping versus flapping with body rotation are nearly identical, with the strain difference between these two cases approximately three orders of magnitude smaller than the strain produced by flapping alone (figure 2*a*,*e*; wings matched for average stiffness with otherwise identical properties). The spatial profiles of strain over the surface of the wing are different for uniform and gradient stiffness wings, with particularly unique spatial profiles when considering the difference between flapping and flapping with rotation (figure 2*b*,*f*). When sensors are optimized for the uniform stiffness wing, rotation detection accuracy is only slightly above chance performance (figure 2*c*,*d*). However, for the gradient stiffness wing, rotation can be detected by optimally placed sensors with 100% accuracy (figure 2*g*,*h*). Note that spike timing changes caused by the addition of rotation are generally relatively small (e.g. orange and purple spike times in figure 2*g*). Optimal sensors are not necessarily found at the locations with the overall greatest strain differences between the two conditions over the course of the wingbeat; rather, locations with large strain differences *at the typical time of spiking* are likely to result in the best ability to distinguish these two cases.

**Figure 3.**
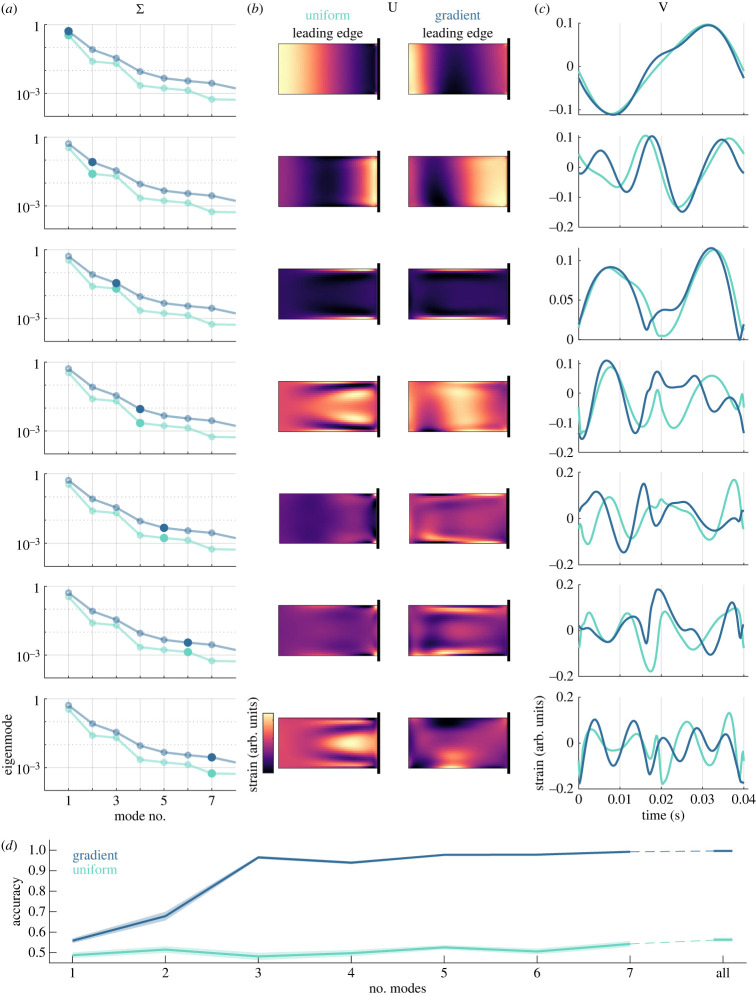
Modal analysis of wing strain shows that few modes are needed to distinguish results in wings with different stiffness gradients. (*a*) Eigenvalue spectra reflecting the relative contributions of each mode to the total strain for wings undergoing flapping without rotation. (*b*) Spatial strain modes. Strain modes are normalized and therefore displayed in arbitrary units (arb. units). (*c*) Temporal strain modes. (*d*) Accuracy as a function of the number of modes used to characterize wing strain. Shaded regions indicate SEM over 20 iterations.

To examine which mechanisms may underlie the greater accuracy observed for wings with nonuniform stiffness, we conducted a modal analysis of the spatio-temporal strain profiles for each wing. This allowed us to identify differences in spatial patterns of strain and temporal patterns of these spatial modes over the course of each wingbeat for uniform versus gradient stiffness wings (figure 3*a*–*c*). Modes with larger eigenvalues (figure 3*a*) contribute more significantly to the full spatio-temporal patterns of wing bending. As expected, spatial asymmetries can be seen for the gradient stiffness wing across all modes, particularly between the leading and trailing edge. Spatial and temporal modes are nonetheless relatively similar across both wings for the first three modes and become more clearly distinct for the fourth mode and above. We assessed classification accuracy as a function of the number of modes used to reconstruct wing strain (i.e. mode 1 only, modes 1–2, modes 1–3, etc.; figure 3*d*). Despite the apparent similarity of the first several modes for uniform and gradient stiffness wings, we found measurable differences in accuracy even for a single mode and maximal differences in accuracy with only three modes. These results demonstrate that the improved performance of nonuniform stiffness wings arises from fundamental differences in wing strain produced by the wing structure, apparent even in lower-order approximations of spatio-temporal strain. Differences in performance do not depend on subtle differences in wing strain captured by higher-order modes, which may be more influenced by small parameter changes or numerical errors from the FEM.

**Figure 4.**
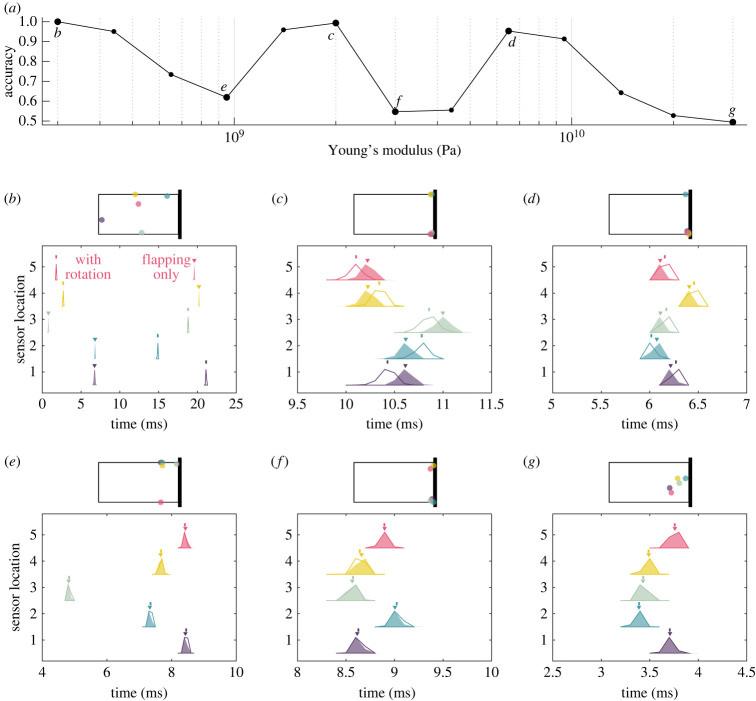
Changes in spike timing underlie non-monotonic changes in accuracy as a function of stiffness. (*a*) Accuracy of pitch detection as a function of wing stiffness for an underdamped wing of uniform stiffness. (*b*–*g*) Spike timing histograms for the top five sensors, with locations indicated by dots of corresponding colours in the above wing schematic (five shown for simplicity, although 10 sensors are used to determine classification accuracy). For each sensor, solid distributions show spike timing on wingbeats with flapping only, with triangles indicating the distribution mean. Distributions with outline only show spike timing on wingbeats with rotation, with vertical lines indicating the distribution mean. Greater differences in spike timing histograms between the two conditions (solid compared with outline) result in greater classification accuracy.

We further investigated how the observed changes in wing strain translate to changes in the timing of sensor responses underlying classification (figure 4). In the examples shown, only the average wing stiffness is varied while all other properties remain the same. The most flexible wing (*b*) exhibits large differences in spike timing between conditions: flapping only (solid) versus flapping with rotation (outline). This leads to 100% detection accuracy because the condition can be perfectly determined based on the spike timing in the optimally placed sensors shown (i.e. the spike timing distributions are non-overlapping for the two conditions). A relatively small change in mean wing stiffness, however, results in large changes in both optimal sensor placement and spike timing, which in turn dramatically reduces detection accuracy (*e*). In this case, spike timing distributions are nearly entirely overlapping between the two conditions, resulting in near-chance accuracy. On the other hand, wings with large differences in stiffness can exhibit similar accuracy (*b*, *c* and *d*). Spike timing occurs at different phases of the wingbeat in each of these cases (even for similarly placed sensors, as in *c* and *d*), yet all result in high classification accuracy due to the distinction between the two distributions (solid and outline). These complex changes in optimal sensor placement and spike timing have the overall effect of producing a non-monotonic relationship between detection accuracy and average wing stiffness (figure 4*a*).

**Figure 5.**
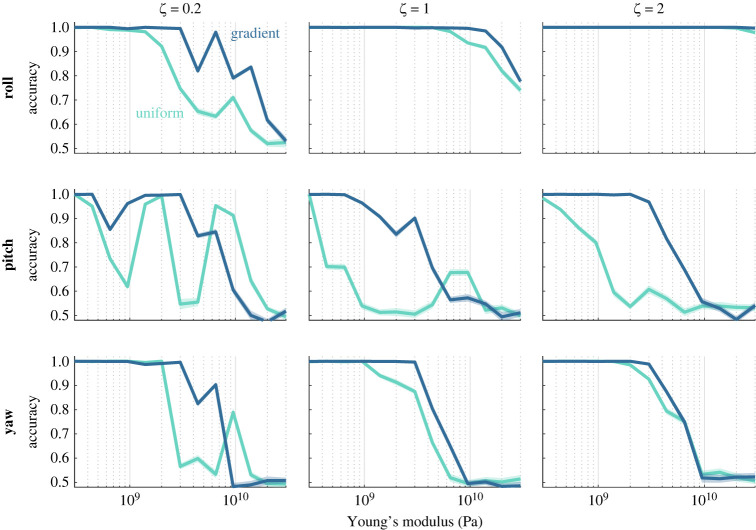
Wings with a stiffness gradient typically result in higher classification accuracy compared with uniform stiffness wings matched for average stiffness. Each panel shows classification accuracy for groups of 10 optimally placed sensors as a function of average wing stiffness. Different subpanels show results for under-, critically and overdamped cases (columns) and for rotation about different axes (rows). For each condition (damping, axis of rotation, average stiffness, uniform or gradient) sensor optimization was performed for 20 separate iterations to assess classification accuracy. Shaded regions show SEM.

We next compare detection accuracy for wings with different stiffness gradients across a range of average stiffness values, different damping ratios and three different axes of rotation (figure 5). For wings matched on other characteristics (i.e. mean stiffness and damping), gradient stiffness wings generally outperform uniform stiffness wings. This trend holds true for almost all damping ratios and axes of rotation for almost all average stiffness values tested. Further, wings with higher damping ratios (i.e. overdamped wings) generally result in higher accuracy when other characteristics are held equal. Accuracy typically decreases as average stiffness increases, although the relationship may be non-monotonic (e.g. pitch detection for a uniform stiffness wing with damping ratio 0.2, also shown in figure 4). Lower damping ratios tend to produce more irregular relationships between accuracy and average stiffness in addition to generally lower accuracy.

**Figure 6.**
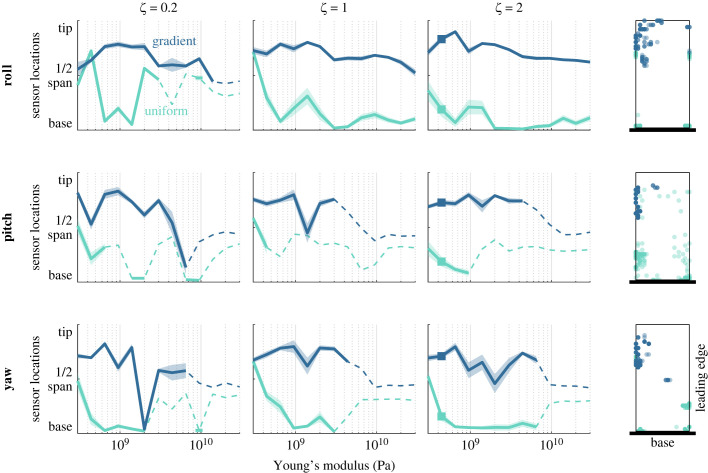
Wings with a stiffness gradient result in optimal sensors placed more distally (towards the wing tip) compared with uniform stiffness wings matched for average stiffness. Average spanwise location (from wing base to wing tip) of 10 optimal sensor locations is shown as a function of average wing stiffness. Shaded regions show SEM. Solid lines show results when average classification accuracy (figure 5) exceeds 70%. Dashed lines show when accuracy falls below this threshold. Note that sensor placement becomes random as classification accuracy approaches chance performance. Right column shows the distribution of optimal sensor locations (10 sensors for each of 20 iterations) for example cases indicated by squares in *ζ* = 2 panels. (See electronic supplementary material, figures S8–S10, for additional examples of detailed sensor placement.)

Previous work showed that optimal sensor placement shifted between locations clustered at the wing base and locations clustered at the wing tip, depending on wing average stiffness and the axis of rotation to be detected [16]. However, these observations were limited by the wing model it used, which not only studied wings of uniform stiffness but also restricted the wings to take on linear combinations of characteristic modal shapes. The direct method in finite element models we employ in the current work, however, is not concerned with mode truncation and allows more complex solutions. Therefore, we next examine how the average spanwise locations of optimally placed sensors depend on features of wing structure (figure 6). Like previous work on uniform stiffness wings, sensors are typically localized near the wing base across a range of other parameters, although as average stiffness decreases, sensors shift distally. On gradient stiffness wings, on the other hand, optimal sensors are found far more distally—generally at intermediate locations on the wing. Note that the stiffness gradient for these wings runs from the leading edge of the wing base (stiffest at the bottom right corner as depicted) to the trailing edge of the wing tip (most flexible at the top left corner), i.e. diagonally. On a given wing, optimal sensors are generally not placed along the line of equal wing stiffness.

**Figure 7.**
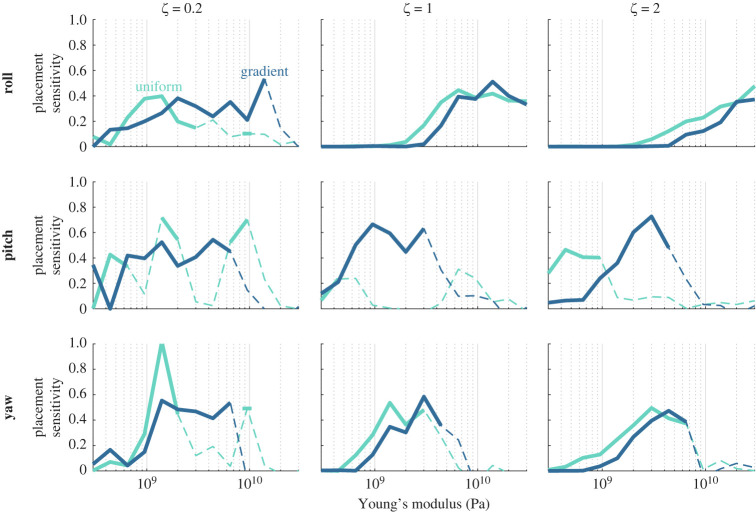
Uniform stiffness wings require more careful sensor placement than wings with a stiffness gradient. Each panel shows classification accuracy of 10 optimized sensors divided by accuracy of 10 randomly placed sensors minus 1 as a function of average stiffness. A value of 1 indicates maximum sensitivity to sensor placement: optimized sensors can achieve perfect accuracy whereas random sensors perform at chance. A value of 0 indicates no sensitivity to sensor placement: random sensors perform equally as well as optimized sensors. Solid lines show results when average classification accuracy (figure 5) exceeds 70%. Dashed lines show when accuracy falls below this threshold.

We have shown that stiffness gradients and overdamped wings generally have sensing advantages across a range of other wing properties and detection tasks for *optimally placed* sensors. However, insects need not have sensory structures optimally positioned, and even on robotic systems, sensor placement may be constrained by other design demands. To examine how closely sensing accuracy depends on optimal sensor placement, we next consider how much classification accuracy suffers when sensors are placed randomly (figure 7). To do so, we devised a measure of *placement sensitivity* based on the ratio of classification accuracies for optimized versus randomized sensor locations: the classification accuracy of 10 optimized sensors divided by the accuracy of 10 randomly placed sensors minus 1. This results in values ranging from 0 to 1, where 0 indicates no sensitivity to sensor placement (i.e. random sensors perform equally as well as optimized sensors), and 1 indicates complete sensitivity (i.e. random sensors perform at chance when optimized sensors perform with perfect accuracy). Accuracy in stiffer wings is typically more sensitive to sensor placement. This is consistent with the general decrease in accuracy observed for these wings: as wings become stiffer, the classification task becomes more difficult, resulting in decreased detection accuracy and increased reliance on particular sensor placement. Although relatively similar across a range of other parameters, placement sensitivity tends to be modestly higher for uniform stiffness wings compared with gradient stiffness wings, indicating that gradient stiffness wings allow for more flexible sensor placement across a range of other wing properties and sensing tasks.

## 4. Discussion

We examine how the structural properties of a wing impact its ability to sense body rotations during flight using finite element models. Our results point to two general conclusions regarding sensing performance for flapping wings: those with a spatial gradient in stiffness and those with a higher damping ratio enable more accurate detection of body rotations. Wings with a stiffness gradient exhibit higher detection accuracy across a range of average stiffness values, damping ratios and across rotation axes (figure 5). Additionally, wings with gradient stiffness are less sensitive to particular sensor placement, with higher detection accuracy for randomly placed sensors relative to that for uniform stiffness wings (figure 7).

### 4.1. Limitations of the current work

There are several limitations and simplifying assumptions in the current work that merit discussion and suggest potential areas of future inquiry. We choose to employ finite element models, which allow great flexibility to investigate the effects of complex wing structures. This increased scope of wing shapes and material properties that we can explore, however, comes with the potential for issues surrounding numerical precision and instability. We conducted two separate convergence studies to mitigate potential numerical issues (electronic supplementary material, figure S4). To provide consistency with prior studies on sparse sensor placement in wings [15,16], we retained the highly simplified rectangular wing shape of earlier models but allowed for more complex spatial patterns of stiffness. However, we are acutely aware that wing morphology—namely, shape and venation pattern—will have significant impacts on the patterns of spatio-temporal strain induced by wing flapping and will, therefore, have significant impacts on sensing accuracy and optimal sensor placement as well. Future work should, therefore, address the effects of different wing morphologies on sensing. Recent work has provided new quantitative tools that bring together data on wing shape and other aspects of wing structure in hundreds of different species [38]. Combining this rich dataset with the framework established here can be used in future work to study the effects of more realistic wing shapes on sensing performance.

This work does not address the potential impacts of aerodynamic forces on wing deformation and thus sensing in wings. This simplifying assumption is supported by previous work showing that aerodynamic forces have minimal impact on wing bending relative to inertial-elastic forces for *Manduca sexta* [8,39]. While COMSOL does not easily allow for simulations of fluid–structure interactions, a series of simulations exploring this issue using vortexlet models for two-dimensional flapping wings shows that, in general, wing bending is indeed relatively unaffected by aerodynamic loads [8]. While the influence of the dynamics of wing bending on aerodynamic forces is considerable, the relative importance of fluid loading on that bending is negligible, except perhaps near resonant frequencies. There are, additionally, other fluid–structure studies of insect wings that have even higher fidelity than afforded by prior vortexlet models. For example, work using a Navier–Stokes solver coupled with structural models of wings points to the importance of structural mechanics on aerodynamic force production, although the study does not address how aerodynamic loads relative to inertial-elastic forces influence wing bending [6]. Nevertheless, aerodynamic forces may more significantly impact bending for smaller wings. Work in several dipteran species has shown that aerodynamic and inertial forces acting on the wing are of a similar order of magnitude [40]. Additionally, even relatively small changes in wing bending may be behaviourally significant if they improve an animal’s ability to sense relevant features of wing bending. Without a full fluid–structure interaction model, it is difficult to determine how aerodynamic loads may influence both the optimal sensor locations and the accuracy of those sensors in detecting perturbations to body dynamics. While such an approach would be computationally intensive, it does represent an interesting future research direction.

Several additional simplifying assumptions were made about how sensors encode information about wing strain and how information from sensors is decoded. For example, our sensors encode information about spanwise normal strain only. Previous work showed that this resulted in better performance than sensors that encode chordwise strain [16]. Additionally, this previous work showed that sensing performance was not improved with more complicated decoding schemes than that used in this work (LDA). As ongoing work uncovers more detail about which features of wing bending biological mechanosensors on insect wings are sensitive to, additional theoretical study may reveal the significance of these response properties.

Due to the nature of these simplifying assumptions, we do not expect that optimal sensor placements found in the current work would correspond directly to sensor placements found on actual insect wings. Simplifications in the wing and neural encoding models as well as assumptions about what is being optimized for (i.e. detection of rotation in the current work) will necessarily lead to discrepancies between optimal sensor placement in our work and optimal sensor placement on insect wings. Moreover, sensing in a biological system need not be optimal but rather may simply be sufficient to meet an organism's needs. It is, therefore, not surprising that in the current work we see optimized sensor placements in qualitatively different locations than typically found on insect wings (e.g. concentrated towards the trailing edge). Nevertheless, the current work shows consistent sensory advantages conveyed by nonuniform stiffness wings. Among these advantages is less sensitivity to sensor placement, supporting the idea that sensors in less optimal but more biologically realistic locations will provide near-optimal sensing performance.

### 4.2. Implications for insect flight

Despite these limitations, the current work nevertheless presents several general results with strong implications for sensing during flapping flight. For example, insect wings exhibit stiffness gradients from wing base to tip and leading to trailing edge [17]. The aerodynamic consequences of wing stiffness have been studied extensively, although most work has focused on wings of uniform stiffness. The results are somewhat mixed, with some studies finding advantages for rigid wings and others showing advantages for flexible wings [4,6,41–44]. Among studies that have specifically considered wings of nonuniform stiffness, and particularly wings that reflect the stiffness gradients observed in actual insects, these wings have generally exhibited a number of advantages over uniform wings, including significant improvements in the production of both lift and thrust forces in flapping wings and flutter-induced drag reduction [5,11,45]. Our results suggest that an additional function of this stiffness gradient may be to aid sensing performance, by directly improving accuracy and/or by allowing for more flexible placement of sensors on the wing. Further, gradient stiffness wings exhibit smoother changes in accuracy as average stiffness changes. This may provide robustness to changes in wing stiffness that occur over an insect’s lifespan [46] and may prevent the need for evolutionary fine-tuning of wing stiffness for sensing purposes. Thus, the current work suggests that aerodynamic and sensing needs may both impart evolutionary pressures towards the observed stiffness gradients of insect wings.

We show that increased damping generally improves sensing performance, while also increasing the smoothness of accuracy as a function of wing stiffness. Insect wings have previously been reported to be underdamped [47,48], although other work has reported that wing veins in particular are overdamped [49]. Our own measurements in *Manduca sexta* wings show a combination of underdamped and overdamped properties (electronic supplementary material, figure S11). Greater damping may help with flight stability, as wing movements will be less affected by external perturbations (such as gusts or collisions) common during flight. Damping may also avoid significant aerodynamic flutter and wing failure. More work is needed to elucidate the consequences of the damping properties observed in insect wings.

Previous work on sensing during insect flight has largely focused on the halteres, the modified hindwings of flies that are generally considered to serve as gyroscopic sensors of Coriolis forces [50–52]. In the current work, we draw on previous studies showing a key sensory function for wings [2,30] and similarly consider how wings may serve this function, detecting small changes in strain induced by constant-velocity rotations. Yet for an insect in flight, sensing transients—induced by gusts or changes in direction, for example—is likely even more behaviourally relevant, yet has not been considered for sensing by either halteres or wings, largely due to the challenges of studying these transients in both experiments and numerical simulations. The transient changes in wing strain induced by these perturbations are much larger than those induced by constant rotation, and thus likely present an easier sensing task. This represents a valuable, if challenging, area of future research due to its likely behavioural relevance.

The structure of insect wings is presumably shaped by many potentially competing demands acting on their evolution. Factors likely include force generation for flight, sensing, robustness to damage, mating display and predator avoidance, among others [53–58]. Perhaps one of the most promising but challenging areas of future study will investigate the interplay between the many competing evolutionary pressures shaping the evolution of insect wings and flight control.

### 4.3. Implications for engineered systems

Much like insect wings, engineered wings for flapping-wing micro aerial vehicles (MAVs) do not have uniform structural properties, and the methods proposed in this work are likely relevant for similar classification tasks like quickly sensing disturbances and rotations in these small vehicles (e.g. [59–63]). Even though these vehicles can often be equipped with an inertial sensor that can measure rotation rates, wing deformation has been predicted to precede body rotation and could provide an earlier warning of unexpected rotations [64]. Beyond flapping flight, this sensing and sensor placement approach may be useful in other engineered systems in which event timing can change when disturbances are applied. Legged robots are another example of cyclic systems that need to quickly classify and react to disturbances (e.g. [65]). For example, an unexpected rotation could alter the pressure on the robot foot within a gait cycle.

Given that the classification approach described above depends on spike timing within a cycle, the work in this paper is particularly relevant to systems with cyclic behaviours. However, the ability to match a sensing system to the synthetic structure and perturbations of interest can likely be extended to non-cyclic engineered systems. Large arrays of sensors have previously been designed for fixed-wing MAVs to gather data on wing deformation during flight with the goal of classifying disturbances before the MAV’s inertial sensor can detect movement [37]. The results of the current study indicate that sensing disturbances like gusts may be facilitated by material choices and stiffness gradients throughout an MAV wing. Similarly, work in the field of soft robotics has shown how useful graded and anisotropic material properties can be for actuation and locomotion [66], but these variations in material properties may also facilitate a robot’s ability to sense unanticipated contact or other disturbances.

Finally, this work suggests an entirely new approach towards sensing than is currently used in engineered systems. The vast majority of engineered systems use sensors that provide an analogue or digital value when sampled. Instead, as shown in figure 2, our approach uses differences in event timing (e.g. a probabilistic crossing of a threshold) to distinguish rotations that would otherwise be exceedingly challenging to sense with conventional strain sensors. Even the simplest off-the-shelf microcontrollers can distinguish digital events down to sub-microsecond resolution, and recent work has shown several 'switch’-based sensors that may be used in a sensing framework as proposed in this paper (e.g. strain [67], acoustic [68], flow [69]). This approach may provide a step towards low-latency and event-based sensing in engineered systems.

## Data Availability

A COMSOL file that implements the FEA model as well as multiple example simulation results are available from the Dryad Digital Repository: https://doi.org/10.5061/dryad.fxpnvx0wq [24]. MATLAB code that implements strain encoding in neural-inspired sensors and sensor placement optimization can be found at https://github.com/aiweber/optimal_sensing_FEAwing. Supplementary material is available online [70].
